# Raman Spectroscopy: Guiding Light for the Extracellular Matrix

**DOI:** 10.3389/fbioe.2019.00303

**Published:** 2019-11-01

**Authors:** Mads S. Bergholt, Andrea Serio, Michael B. Albro

**Affiliations:** ^1^Centre for Craniofacial and Regenerative Biology, King's College London, London, United Kingdom; ^2^Department of Mechanical Engineering, Boston University, Boston, MA, United States

**Keywords:** label-free imaging, Raman spectroscopy, extracellular matrix, collagen, glycosaminoglycans, fiber-optic diagnostics, tissue engineering

## Abstract

The extracellular matrix (ECM) consists of a complex mesh of proteins, glycoproteins, and glycosaminoglycans, and is essential for maintaining the integrity and function of biological tissues. Imaging and biomolecular characterization of the ECM is critical for understanding disease onset and for the development of novel, disease-modifying therapeutics. Recently, there has been a growing interest in the use of Raman spectroscopy to characterize the ECM. Raman spectroscopy is a label-free vibrational technique that offers unique insights into the structure and composition of tissues and cells at the molecular level. This technique can be applied across a broad range of ECM imaging applications, which encompass *in vitro, ex vivo*, and *in vivo* analysis. State-of-the-art confocal Raman microscopy imaging now enables label-free assessments of the ECM structure and composition in tissue sections with a remarkably high degree of biomolecular specificity. Further, novel fiber-optic instrumentation has opened up for clinical *in vivo* ECM diagnostic measurements across a range of tissue systems. A palette of advanced computational methods based on multivariate statistics, spectral unmixing, and machine learning can be applied to Raman data, allowing for the extraction of specific biochemical information of the ECM. Here, we review Raman spectroscopy techniques for ECM characterizations over a variety of exciting applications and tissue systems, including native tissue assessments (bone, cartilage, cardiovascular), regenerative medicine quality assessments, and diagnostics of disease states. We further discuss the challenges in the widespread adoption of Raman spectroscopy in biomedicine. The results of the latest discovery-driven Raman studies are summarized, illustrating the current and potential future applications of Raman spectroscopy in biomedicine.

## Introduction

### The Extracellular Matrix

The extracellular matrix (ECM) is a fundamental component of all biological tissues. The ECM consists of complex, integrated networks of extracellular biomolecules that serve to provide tissues with structural and biochemical support. ECM structures give rise to a multitude of tissue-specific functionalities, providing mechanical stability, molecular transport barriers, junctions for cell adhesion, transduction of mechanical cues, and reservoirs of growth factors. The ECM is composed of a myriad of biomolecular constituents, including fibrous proteins (e.g., collagen, elastin), glycoproteins (e.g., laminin, fibrillin, fibronectin), glycosaminoglycans (GAG) (e.g., chondroitin-sulfate, heparin, hyaluronan), GAG-rich proteoglycans (e.g., aggrecan, versican, decorin), and lipids (e.g., cholesterol, phospholipids). These constituents are organized in unique, tissue-specific arrangements that are optimized in terms of content, distribution, and alignment to achieve requisite structure-function relationships. For instance, musculoskeletal connective tissues consist of collagen networks interspersed with dense proteoglycans, giving rise to requisite combinations of tensile and compressive mechanical resistance (Chahine et al., 2004) and low friction interfaces (Krishnan et al., 2003). In the cardiovascular system, the arterial wall is composed of an interwoven network of elastic and collagenous fibers, providing vessels with an important combination of pulsatile energy efficiency and rupture strength (Cocciolone et al., 2018). In the nervous system, lipid-rich myelin sheaths surround and insulate neurons, allowing for effective long-range transmission of electrical impulses. Ultimately, the make-up and organization of the ECM is vital for the functionality, health, and longevity of biological tissues. Furthermore, pathological tissue disorders are typically associated with the disruption of or abnormalities to the ECM (Bonnans et al., 2014). Prominent examples include: (i) fibrosis from aberrant matrix protein secretion, as characterized in pathology of vital organs [e.g., lung (White, 2015), kidney (Duffield, 2014), heart (Prabhu and Frangogiannis, 2016), and liver (Kisseleva, 2017)], (ii) musculoskeletal tissue degeneration from the break-down of the load-supporting ECM (Krishnan and Grodzinsky, 2018), and (iii) metastatic cancers from the invasion of malignant cells through ECM barriers (Gilkes et al., 2014). Accordingly, the ability to characterize the make-up and organization of the ECM is critical for understanding the onset of pathologies and for developing novel disease-modifying therapeutics.

### Imaging of the Extracellular Matrix

Imaging techniques, such as conventional microscopy, have long served as tools for studying the ECM. Today, characterizations of the ECM continue to rely heavily on conventional histology staining techniques, such as colorimetric dyes for general ECM constituents (e.g., Alcian Blue and safranon O for GAG, Picrosirius Red for collagen) and colorimetric or fluorescent-conjugated antibodies for imaging specific molecular ECM constituents. While these methods offer detailed and specific characterizations of protein distributions, they are associated with a host of well-documented limitations (Hyllested et al., 2002), such as sample fixation artifacts (Pousty et al., 1975; Wilson and Gardner, 1980), non-stoichiometric dye binding (Puchtler et al., 1988), and lack of dye binding specificity (Scott and Stockwell, 1967; Jubb and Eggert, 1981), which limit their ability to provide reliable quantitative or even semi-quantitative assessments of tissue ECM distributions. Further, histological staining is limited by its association with highly laborious sample preparation protocols. To complement and potentially advance beyond conventional staining techniques, microscopy is continually evolving, enabling novel imaging capabilities from the tissue-scale to nano-scale level.

A palette of imaging technologies has been used for characterizations of the ECM, including widefield fluorescence microscopy, confocal microscopy, polarized light microscopy (PLM), and super-resolution microscopy (Brightman et al., 2000). Moreover, a number of advanced, non-linear, label-free imaging modalities for studying the ECM and cell-ECM interactions have been developed, including second harmonic generation (SHG) microscopy (Burke et al., 2013), two-photon excited fluorescence (TPEF) microscopy and terahertz (THz) spectroscopy imaging (Jung et al., 2012a,b; Stantchev et al., 2018). These imaging techniques provide the advantage of offering detailed insights into the structural characteristics of tissue ECMs. Further, ultrastructural imaging can be archived using scanning electron microscopy (SEM) (Santi et al., 2016) or atomic force microscopy (AFM) (Jorba et al., 2017), while specific compositional imaging can be achieved with complementary mass spectrometry (Gessel et al., 2015). A range of optical spectroscopy techniques, such as Fourier transform infrared (FT-IR) spectroscopy (Cheheltani et al., 2014), have also been extensively applied to characterize the ECM. FT-IR measures the vibrational absorption spectrum of molecules and has been used to elucidate the distribution of collagen in sectioned tissues (Spalazzi et al., 2013). State-of-the-art FT-IR microscopic imaging using focal plane array (FPA) detectors offers rapid snapshot spectral imaging for biomolecular analysis. FT-IR, however, exhibits several limitations, such as relatively low imaging resolution and incompatibility with imaging aqueous ECM tissue environments.

Raman spectroscopy is an inelastic light scattering technique that offers a unique potential for advanced ECM characterizations. Raman scattering occurs when light induces a change in the polarizability of molecules. In recent years, the use of Raman spectroscopy in biomedical applications has received growing attention due to its ability to achieve robust quantitative assessments of the biochemical composition of biological tissues. Raman spectral peaks are highly specific to molecular chemistry (Shetty et al., 2006) with intensities that are proportional to molecular content (Bergholt et al., 2017), allowing for critical assessments of both the ratios and concentrations of individual ECM constituents in a specimen. Further, Raman spectral acquisitions do not require molecular labeling or contrast agents, allowing for non-invasive or minimally-invasive ECM assessments. Two rapidly expanding applications for Raman spectroscopy in biomedicine are: i) Raman spectroscopic imaging (Raman microscopy) for characterizing the make-up and spatial distribution of ECM constituents throughout biological tissue sections, and ii) fiber-optic based Raman spectroscopy for diagnostics of ECM alterations in the *in vivo* environment. For microscopy-based applications, Raman spectroscopy is compatible with hydrated tissues and can yield images with diffraction-limited spatial resolution, allowing for the generation of high resolution quantitative images of the ECM distribution in live or unprocessed tissue specimens. Fiber-optic based diagnostics benefit considerably from the label-free nature of Raman acquisitions, allowing for minimally invasive quantifications of critical ECM alterations that are associated with disease states. Overall, Raman spectroscopy is now widely applicable for an extensive range of *in vitro, ex vivo*, and *in vivo* ECM-related characterizations and diagnostics. These developments have occurred alongside the establishment of advanced computational methods, including multivariate algorithms, spectral unmixing, and machine learning approaches in order to extract and characterize the ECM tissue structure and composition at the molecular level. These computational methods have greatly aided the development of Raman spectroscopy ECM characterizations in the areas of imaging and diagnostics.

In this article, we review the role and applications of state-of-the-art Raman spectroscopy for ECM characterizations. The results of the latest Raman microscopy imaging and fiber-optic diagnostic techniques are summarized, spanning from regenerative medicine assessments to disease diagnostics, and illustrating both current and potential future applications in biomedicine.

## Raman Spectroscopy

**Figure 1:**
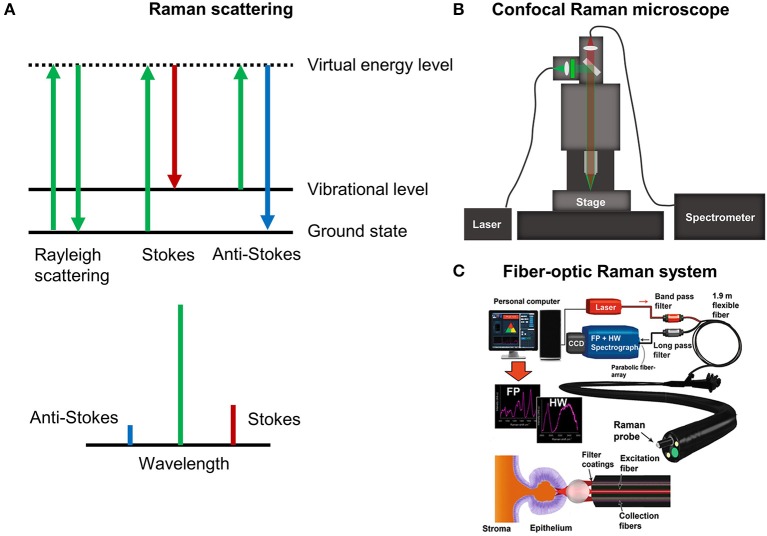
**(A)** The Raman effect. **(B)** Schematic of confocal Raman microscopy platform for imaging. **(C)** Example of fiber-optic Raman spectroscopy for endoscopy measurements in the gastrointestinal tract [Reprinted with permission from Bergholt et al. (2016b)].

Conventional (spontaneous) Raman scattering is an inelastic interaction between light and molecules. When light interacts with a molecule, it can be excited to a short-lived virtual state that immediately falls back to a vibrational excited state in the electronic ground state (Figure 1A). Because of this interaction, a small amount of energy is transferred or removed from the molecule and the resulting scattered light is red shifted (stokes) or blue shifted (anti-stokes) containing encoded vibrational molecular information (i.e., fingerprints). For this reason, Raman scattering of tissues offers a wealth of information about the vibrational structure of their compositional proteins, GAGs, lipids, and DNA. Raman spectra are often recorded in the so-called fingerprint region (400–1,800 cm^−1^) that contains relatively weak but highly specific Raman peaks, allowing for ECM assessments with a remarkably high degree of biomolecular specificity. Recently, additional attention has been drawn to the use of the high wavenumber region (2,800–3,600 cm^−1^), which contains Raman bands that are less specific but exhibit a higher degree of signal intensity.

### Raman Spectroscopy Instrumentation

Raman spectra of tissues can be measured using a microscope or custom fiber-optics. A state-of-the-art confocal Raman microscope is shown in Figure 1B. Briefly, the laser is coupled into the microscope using a single-mode fiber and illuminated onto the sample with a microscope objective. Raman spectroscopic-based confocal imaging can be achieved by collecting the backscattered light using a fiber. The single fiber acts as pinhole and couples the light into a high-throughput spectrometer that disperses it onto a charge coupled device (CCD) camera. A valuable growing application of Raman microscopic imaging is the generation of hyperspectral Raman images, whereby spectra are acquired at discrete positions over the surface or exposed cross-section of a specimen and analysis is performed to generate a spectral-based image. For these applications, rapid raster scanning is typically performed using a motorized or piezoelectric stage. Despite the traditional shortcoming of weak intensity tissue signals, rapid advances in instrumentation and detectors have improved the speed at which Raman spectral images can be measured, reducing the acquisition time of high resolution images to only hours or minutes. Several variants of Raman microscopy systems exist, such as confocal Raman microscopy (Puppels et al., 1990), light sheet Raman microscopy (Oshima et al., 2012), and line scan Raman microscopy (Grauw et al., 1997). In general, fiber-based confocal microscopy setups offer superior imaging of the ECM compared to conventional non-confocal setups, in terms of Raman signal quality. This can be attributed to the rejection of out-of-focus scattered light. Integration times for measuring Raman images of tissues can be as low as 0.1 s for a 532 nm high numerical aperture (NA) confocal Raman setup. Hence, a full hyperspectral image (e.g., 100 × 100 pixels) can be measured within ~20 minutes. Single Raman spectra of tissues can also be measured using fiber-optic sampling. Figure 1C shows an example of an endoscopic compatible Raman platform used for the *in vivo* tissue analysis of colon cancer. The laser light is coupled into a 2 mm bifurcated fiber-optic probe that delivers the laser and collects the Raman scattered light within 1 s, which can then be analyzed using computational methods in real-time.

The choice of laser excitation wavelength is one of the most important parameters when performing Raman spectroscopy. Generally, wavelengths are selected based on a compromise between reducing background autofluorescence and maximizing Raman signal intensities, both of which increase with decreasing wavelength (the Raman signal scales with the 4th power of the excitation frequency). The optimization of this balance is highly dependent on the sample type and application. Most tissues will give sufficient Raman signal and desirable low autofluorescence under near-infrared (NIR) 785–1,048 nm laser excitation (Lim et al., 2011; You et al., 2017). There are exceptions, as some tissues can exhibit prohibitively high autofluorescence even under NIR excitation [e.g., liver, spleen, heart, kidneys, lung tissue (Huang et al., 2011)]. For samples with particularly low degrees of autofluorescence (e.g., cartilage, bone, cell monolayers), it is often advantageous to use shorter excitation wavelengths, such as 633 or 532 nm, which significantly speeds up the acquisition time due to the generation of stronger signals. It is important to consider that Raman spectra obtained with different laser excitation wavelengths should, in general, not be compared directly due to possible molecular resonance effects.

The imaging resolution of Raman microscopy depends on the specific type of system but is ideally diffraction-limited and, therefore, depends heavily on the excitation wavelength and choice of objective. For a state-of-the-art confocal Raman system with visible excitation, the lateral resolution can reach as low as 300 nm and the depth resolution can reach less than 500 nm for sufficiently small pinhole settings.

### Raman Microscopy Sample Preparation

For Raman microscopy-based applications, the compatibility of sample processing techniques must always be considered. A major advantage of Raman spectroscopy is its exceptional compatibility with aqueous environments. As such, imaging can be performed directly on hydrated tissues in buffered saline solution without the implementation of any sample processing (Bergholt et al., 2016a) (e.g., fixation, dehydration, or embedding). For the acquisition of large hyperspectral images, it is often advantageous to analyze specimens with flat surfaces in order to maintain a consistent focus across a large region of interest. Sample flattening can be readily achieved with cryostat trimming or, if the preservation of tissue viability is required, with a vibratome or a template cutting block. Tissues can also be sectioned through conventional histology sectioning protocols (Bergholt et al., 2018). One drawback of Raman imaging is that paraffin embedding is not suitable for Raman acquisitions due to significant spectral interference. Both digital and experimental deparaffinization approaches have been explored (Tfayli et al., 2009; Gaifulina et al., 2016). Tissues can be readily imaged in either a fresh or formalin fixed state, though formalin fixation introduces subtle differences in the Raman spectra due to molecular crosslinking (Huang et al., 2003a; Meade et al., 2010). Further, it is important to note that the use of conventional microscopy glass slides is not suitable for non-confocal Raman spectroscopy and typically requires the use of special substrates [e.g., aluminum, steel, calcium fluoride (CaF_2_) or magnesium fluoride (MgF_2_)] that induce negligible background signal. State-of-the-art confocal Raman microscopy with high magnification objectives and a small pinhole, however, offers imaging with essentially no contribution from conventional glass substrate even for imaging of a monolayer of cells (Kallepitis et al., 2017).

### Raman Spectroscopy Analysis

Due to the large amount of data obtained from hyperspectral Raman images, computational analysis is essential in order to extract molecular information. Before Raman spectra are analyzed, they must be preprocessed in order to reduce confounding factors, such as background autofluorescence and noise. Common methods to do so include cosmic ray rejection, autofluorescence subtraction, dark signal subtraction, and normalization (Lieber and Mahadevan-Jansen, 2003; Zhao et al., 2007; Hedegaard et al., 2016). Further, to improve reproducibility across instruments, standardization of the Raman intensities should, in general, be performed through system response correction using a NIST standards, such as standard reference materials range (e.g., SRM2241 for 785 nm laser excitation). For sufficiently flat samples, semi-quantitative images may be derived from the absolute signal, whereas spectral normalization schemes may be required to achieve artifact-free images for samples with thickness variations. In temporal studies, it is often important to combine Raman images from several different time points in order to perform quantitative image analysis. Due to varying focus of the microscope objective and, therefore, varying absolute intensities, this requires careful standardization of the Raman images, such as through spectral normalization (Hedegaard et al., 2016; Kallepitis et al., 2017).

**Figure 2:**
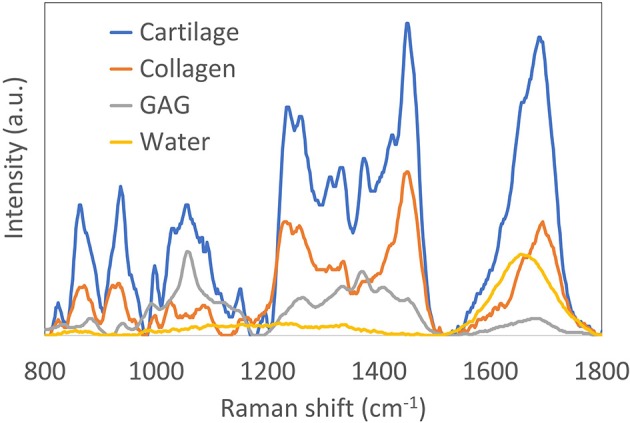
Reprinted and modified with permission from Bergholt et al. (2017). Raman spectra (785 nm excitation) of purified collagen, glycosaminoglycan (GAG) and water. Also shown is the Raman spectrum of articular cartilage, demonstrating the complexity of resolving the individual constituents.

There has been remarkable advancement in the development of computational methods for analysis. Overall, there exist several different approaches to performing spectral analysis of Raman images. The simplest approach is univariate peak analysis by mapping the intensities of a single Raman peak, gaussian peak fitting, or peak intensity ratios. A comprehensive list of tentatively assigned Raman peaks can be found in Table 1. While univariate peak imaging can offer approximations of the visualization of highly concentrated ECM components (Bergholt et al., 2016a), it often yields limited molecular specificity due to the inherent overlapping spectra of individual ECM constituents; Figure 2 shows an example of spectra of purified collagen, GAG, and water, as well as the compound spectrum from native articular cartilage tissue. Alternatively, the implementation of more advanced multivariate or machine learning approaches can be used to extract accurate quantitative information for specific ECM constituents. Multivariate and machine learning offer, in general, more accurate quantitative analysis, as they consider the full range of spectral information. These techniques generally come in two forms: unsupervised and supervised analysis.

**Table 1:** Tentative assignments of Raman peaks of the extracellular matrix and cells.

**Raman peak**	**Tentative assignment**	**Associated molecule**	**References**
785–795	ν_s_(O-P-O)	DNA	Kuhar et al., 2018
820–939	ν(C-C)	Proteins (collagen)	Janko et al., 2010
960	ν(PO_4_)	Hydroxyapatite	Bergholt et al., 2016a
1,002–1,004	ν_s_(C–C)	Phenylalanine of proteins	Lim et al., 2011
1,063	ν_s_(S = O)	GAG	Demers et al., 2015
1,080	ν(C-C)	Lipids	Czamara et al., 2015
1,230–1,280	Amide III	Proteins	Rygula et al., 2013
1,375–1,410	ν_s_(COO^−^)	GAGs	Bergholt et al., 2016a
1,400–1,500	b(CH_2_/CH_3_), sc(CH_2_/CH_3_), b(CH_2_)	Lipids	Kuhar et al., 2018
1,445–1,456	b(CH_2_/CH_3_), sc(CH_2_/CH_3_), b(CH_2_)	Proteins	Lim et al., 2011; Esmonde-White, 2014
1,650–1,680	Amide I υ(C = C)	Proteins	Rygula et al., 2013
1,745	υ(C = O)	Lipids	Movasaghi et al., 2007
2,850–2,854	υ_s_(CH_2_)	Lipids	Czamara et al., 2015; Lu et al., 2015
2,880	υ_as_(CH_3_)	Lipids	Mo et al., 2009; Czamara et al., 2015
2,931–2,940	υ(CH_3_)	Proteins	Mo et al., 2009
3,100–3,600	υ(OH)	H_2_O	Leikin et al., 1997; Lu et al., 2015

*The Raman spectra of various tissue biomolecules are highly overlapping, and the peak position is often reported with variability of ~±1–5 cm^−1^ due to instrument variability*.

### Unsupervised Algorithms

#### Principal Component Analysis

The most commonly used technique is principal component analysis (PCA) (Shetty et al., 2006). PCA finds the orthogonal principal components (PCs) of a dataset that successively account for the largest variability in the dataset. For instance, PCA can be used to reduce the dimension of the spectral data significantly while reducing noise and providing more robust models. In general, PCA can be applied as an exploratory technique for hyperspectral Raman imaging, as it enables easy identification of the largest spectral variability, as well as imaging artifacts. PCA is often combined with linear discriminant analysis (LDA) in supervised settings. A major limitation of PCA is that it does not offer pure estimates of components.

#### Multivariate Curve Resolution (MCR)

This powerful technique is applied to deconvolve a specimen's Raman spectra into the underlying spectra of its dominant ECM constituents through variance in the data (Albro et al., 2018). In contrast to PCA, MCR offers estimates of the concentrations of ECM constituents in a tissue. As a limitation, since the deconvolution analysis is generally ill-determined, MCR can encounter difficulties for the analysis of complex tissues with an excessive number of concentrated ECM components or for experiments with insufficient sized data sets. A good practice to validate the modeling capability of MCR is, therefore, to correlate the MCR components with Raman spectra of purified or commercially available components.

#### Clustering Analysis

Another type of unsupervised analysis is clustering techniques, such as *k*-means or nearest neighbor clustering. In k-means clustering the spectra are partitioned into *k* clusters where each spectrum belongs to the cluster with the nearest mean. These exploratory techniques can be used to group similar spectra and can give insights into tissues that have similar or different composition (Kong et al., 2013).

### Supervised Algorithms

#### Partial Least Squares (PLS)

Partial least squares (PLS) represents a regression technique like PCA, but the latent variables (LVs) are further rotated to correlate with reference concentrations. This technique has few applications for Raman imaging, but is better suited for spectroscopic quantification or classification. Cross validation or independent validation should always be used to determine the model complexity. We recommend being highly conservative in choosing the number of LVs in the model to efficiently avoid overfitting the data.

#### Non-negativity Least Squares (NNLS)

Non-negativity least squares (NNLS) is an implementation of regression analysis with an imposed constraint to avoid negative abundances of biomolecules. Regression is typically performed against a library of Raman spectra measured from purified biomolecules (Haka et al., 2006). This allows one to estimate the concentration of the respective biomolecules. In general, care should be taken in regard to NNLS interpretations since these techniques require prior information of ECM components, which may not fully account for the molecular complexity present in the tissue.

#### Spectral Unmixing

Another type of computational analysis is the so called spectral unmixing techniques adapted from satellite imagery. These techniques seek to find the purest pixels in the hyperspectral Raman image using various implementations, including vertex component analysis (VCA) or N-finder (N-FINDR) (Hedegaard et al., 2016). Regression analysis is then applied to these pure spectra in order to extract the abundance of biomolecules in the tissue. Prior information in terms of reference spectra of pure chemicals is advantageous for correlation with the spectral signatures.

#### Classification

For classification purposes, a broad range of machine learning algorithms can be applied. For instance, for diagnostic tissue measurements, techniques such as LDA (Hutchings et al., 2010), partial least squares discriminant analysis (PLS-DA) (Bergholt et al., 2014), support vector machines (SVM) (Widjaja et al., 2008), and classification and regression trees (CART) (Teh et al., 2008) have been used. Many of these algorithms require determination of model complexity and should generally be assessed either through independent cross validation or a validation set.

All the above algorithms can be found in the implementations or inbuilt toolboxes in the Matlab, Python, and R scripting environments.

## Raman Spectroscopy of the Extracellular Matrix

### Cartilage Extracellular Matrix

The organization of articular cartilage ECM is critical for its mechanical functionality. The cartilage ECM consists of collagen fibers interspersed with GAG macromolecules, which vary in concentration and alignment through the depth of the tissue, giving rise to its requisite mechanical and tribological behavior (Helen et al., 1970). During the onset of degenerative conditions, such as osteoarthritis (OA), the cartilage ECM is disrupted, contributing to the progressive degeneration of the tissue (Pearle et al., 2005). As such, OA research has long relied heavily on ECM characterization techniques. Interestingly, due to the strong Raman scattering properties of GAG and collagen, cartilage represents an ideal tissue for Raman spectroscopy applications. As outlined below, prior work on Raman-based cartilage ECM characterizations fall within the categories of (i) Raman microscopy imaging characterizations of the ECM distribution in tissue sections, and (ii) fiber-optic Raman assessments, aimed toward developing clinical intra-articular OA diagnostic platforms (Pavlou et al., 2018).

**Figure 3:**
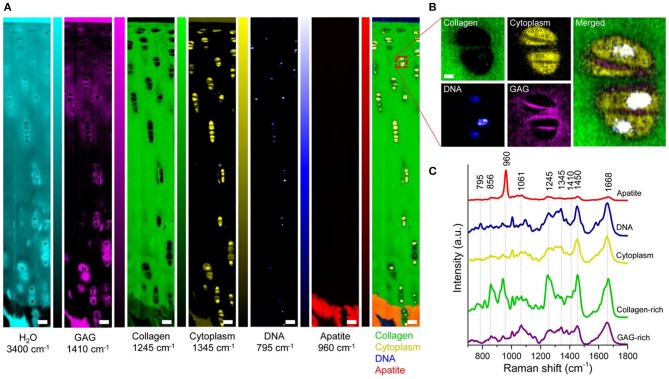
[Reprinted with permission from Bergholt et al. (2016a)]. Raman spectroscopic imaging of articular cartilage. **(A)** Univariate Raman spectroscopy images of articular cartilage showing the band intensity associated with H_2_O (3,400 cm^−1^), GAG (1,410 cm^−1^), collagen (1,245 cm^−1^), cytoplasm (1,345 cm^−1^), DNA (795 cm^−1^), apatite (960 cm^−1^), and the overlay. Scale bar: 50 μm. **(B)** High-resolution (~0.3 μm) Raman spectroscopy image of chondrocytes and pericellular matrix obtained by imaging the GAG (1,410 cm^−1^), cytoplasm (1,345 cm^−1^), and DNA (795 cm^−1^) against collagen (1,245 cm^−1^). Scale bar: 3 μm. **(C)** Representative Raman spectra measured from articular cartilage with marked signatures for specific tissue components.

For work in Raman microscopy ECM characterizations, a recent study utilized NIR excitation to identify Raman bands that represented the presence of proteoglycans in both bone and cartilage tissues (Gamsjaeger et al., 2014). The analysis allowed for imaging of the ECM, yielding the distribution of GAG and type-II collagen in these tissues. Bonifacio et al. (2010) further used 632 nm laser excitation to generate images of the distribution of collagen and GAG across cartilage tissues using both PCA and PLS. In our own study of articular cartilage imaging, we performed confocal Raman microscopy using 532 nm laser excitation to obtain biomolecular and structural insights into the zonal complexity of articular cartilage. Using univariate and multivariate analysis (MCR), we extracted and quantified the collagen, GAG, and water signals of the ECM. Examples of univariate Raman images of bovine articular cartilage cross sections are shown in Figure 3. High resolution imaging showed that it is further possible to image the ECM distribution of the chondrocyte pericellular matrix. This study further demonstrated the advantage of using 532 nm laser excitation in a confocal setup to increase signal and reduce acquisition times, relative to NIR excitation.

Over the past decade, there has been growing interest in the potential applicability of fiber-optic Raman spectroscopy to offer novel advances in OA diagnostics. Given the limitations in current OA diagnostic technologies in terms of precision and instrumentation costs (Hayashi et al., 2016), the development of intra-articular fiber-optic Raman probes can potentially serve as a revolutionary OA diagnostic platform with particular utility in assessments of the critical early stages of the disease. Investigations into this avenue have focused predominantly on initial characterizations of degenerated cartilage on microscopy-based systems, serving as a precursor to the development of more sophisticated fiber-optic platforms required for clinical diagnostics. In an early study, Lim et al. (2011) used a 785 nm Raman microscope to show that impact-injured porcine cartilage explants exhibit changes to the pyranose 1,126 cm^−1^ band intensity and the band area ratio of proline (856 cm^−1^) to hydroxyproline (875 cm^−1^), representing the GAG signal and collagen stability, respectively. Takahashi et al. (2014) performed Raman spectroscopic assessments with 647 nm excitation on human OA cartilage from knee arthroplasty procedures. Results demonstrated a host of peak intensity changes that varied with OA grade, notably an increase in the amide III band ratio of 1,241–1,269 cm^−1^ with OA. Kumar et al. also analyzed human OA cartilage using confocal Raman spectroscopy and demonstrated sensitivity to different grades of the disease (Kumar et al., 2015). Tong et al. (2018) performed Raman spectral characterizations on porcine cartilage explants subjected to mechanical wear regimens, demonstrating a decrease in peak intensities corresponding to the protein backbone and amide bonds in collagen (817 and 1,670 cm^−1^). In an interesting novel analytical approach, Unal et al. (2019) recently demonstrated that analysis of high wavenumber Raman spectroscopy (2,800 to 3,800 cm^−1^) can be used to detect the water fractions in articular cartilage (bound versus unbound water) and the elevated tissue swelling associated with OA - results that were correlated with MRI measurements. In an example of the implementation of fiber-optic Raman spectroscopy, Esmonde-White et al. (2011) demonstrated that a fiber-optic probe can be used to assess cartilage thickness and mineralization degree in cadaver knee joint tissues. Further, de Souza et al. (2014) used a fiber-optic Raman spectroscopic probe to demonstrate increases in Raman ratios related to mineralization and tissue remodeling in an OA-induced rat model. Overall, these investigations demonstrate that definitive spectral signature changes do occur in degenerating OA cartilage, strongly supporting the use of Raman spectral probes for molecular ECM analysis and degenerative diagnostics.

### Bone Extracellular Matrix

Bone is a highly complex tissue comprised of two major structural ECM components: crystals of apatite and collagen fibrils arranged in a hierarchical structure. Raman spectroscopy has extensively been used to analyze how changes in bone composition and structure influence tissue-level bone mechanical properties (Draper et al., 2005; Mandair and Morris, 2015). Raman spectroscopy offers highly detailed information on both the bone mineral as well as the collagen matrix components (Raghavan et al., 2010). Due to the highly crystalline structure of bone, this tissue exhibits very strong Raman scattering. The highly intense phosphate mineral band near ~960 cm^−1^ (ν_1_${\text{PO}}_{4}^{3-}$) is characteristic of carbonated apatites and is the most commonly used marker for bone tissue.

Raman techniques for bone assessment range from microscopy of bone tissue to fiber-optic and computed tomographic (CT) reconstruction approaches (Demers et al., 2012, 2015; Shu et al., 2018). A variety of applications have been reported, ranging from diagnostics of age-related bone loss (Donnelly et al., 2010), osteogenesis imperfecta (Imbert et al., 2014), osteoporosis, and the effect of therapeutic agents (Gamsjaeger et al., 2011, 2013; Olejnik et al., 2014). As an example of recent fiber-optic techniques, Shu et al. developed a spatial offset Raman spectroscopy (SORS) approach in order to non-invasively measure the bone signal *in vivo* from rodents (Shu et al., 2018). The SORS concept is based on diffuse light scattering and the principle that Raman scattered photons originating deep from the tissue can be detected as spatially offset from the excitation light while the shallow Raman photons can be detected closer to the excitation light (Matousek et al., 2005; Matousek and Stone, 2016). For instance in one SORS study on bone strength, Raman spectra were measured from the tibiae of mice aged 4–23 weeks old (Shu et al., 2018). PLS regression was then used to generate predictions of the areal bone mineral density (aBMD), volumetric bone mineralization density (vBMD), and maximum torque (MT) of each tibia as quantified by dual-energy X-ray absorptiometry, microCT imaging, and biomechanical tests, respectively. The authors found significant correlations between Raman spectral predictions and the reference values in all three categories, indicating that the fiber-optic Raman technique has great potential for diagnosing bone diseases, such as osteoporosis.

A range of novel fiber-optic Raman techniques based on diffuse Raman CT techniques has also been developed for transcutaneous imaging (Schulmerich et al., 2008a; Demers et al., 2012, 2015). These techniques have great potential for characterization of bone and tissue *in vivo*. For instance, Schulmerich et al. (2008b) applied a Raman CT to image canine bone tissue. Altogether, Raman spectroscopy represents a particularly powerful approach for bone ECM characterization due to the intense Raman signal originating from the ECM minerals and can therefore be applied across a broad range of applications.

### Cancer Extracellular Matrix

Cancers are associated with abnormalities to the ECM. Destruction of the ECM barriers allow cancer cells to migrate from their *in-situ* location and metastasize systematically. Structural and biomolecular imaging of the ECM is, therefore, particularly important for shedding light on cancer diseases and developing novel therapeutics. Raman spectroscopy and imaging of cancers is an exciting and vibrant field; the examples are numerous and are continuously growing. A large number of studies have used Raman spectroscopy and imaging as an exploratory tool to characterize both the ECM and cells of cancer tissue across many organs, including the oral cavity (Barroso et al., 2016), skin (Kong et al., 2013), breast (Kong et al., 2014), prostate (Aubertin et al., 2018), lung (Huang et al., 2003b), esophagus (Kendall et al., 2003), gastric (Bergholt et al., 2014), and colon (Stone et al., 2004; Brauchle et al., 2018). Common across these studies is that cancer tissue shows a highly distinct spectral signature originating from the ECM and cells. As an example, Raman spectroscopy can be used to monitor how epithelial cells respond to signals from the ECM and, in particular, how cells lose their normal interactions with the ECM during cancer progression (Surmacki et al., 2013). For instance, Paidi used Raman imaging to evaluate pre-metastatic lungs and identified collagenous stroma and its cross-linkers, as well as proteoglycans (Paidi et al., 2017). They associated this with the metastatic potential of the primary tumor by recapitulating the compositional changes in the lungs. The correlated Raman results, histological assessment, and gene expression analysis suggested that remodeling of the extracellular matrix components may present promising markers for objective recognition of the pre-metastatic niche.

### Cardiovascular Extracellular Matrix

**Figure 4:**
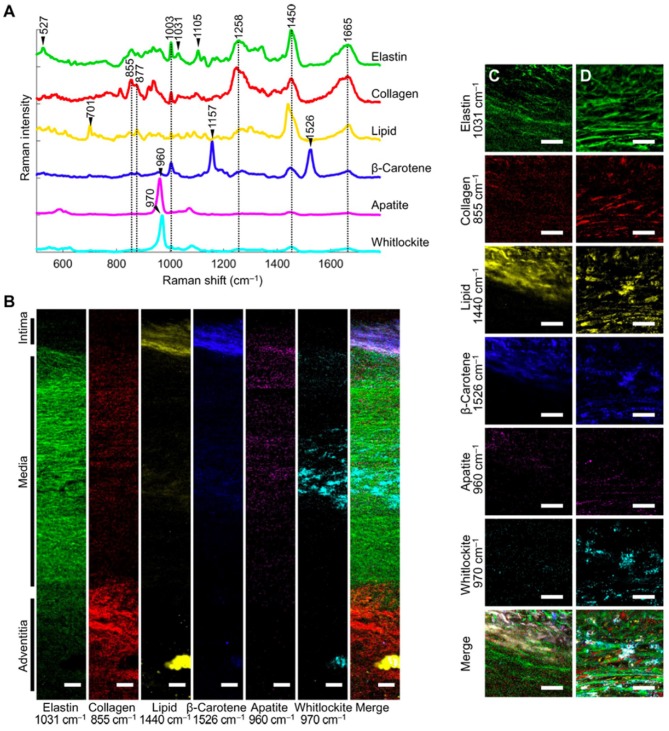
[Reprinted with permission from You et al. (2017)] **(A)** Representative spectra rich in specific aortic components. **(B)** Univariate heat maps of the entire cross section of a non-atherosclerotic aorta were plotted according to the signature peaks listed. **(C,D)** High-resolution maps at the intima-media interface **(C)** and within the media **(D)** were also plotted. Merge refers to a composite image of all the univariate heat maps. Scale bars, 100 μm **(B)** and 50 μm **(C,D)**.

In the cardiovascular system, the arterial wall is composed of a complex network of elastic and collagenous fibers. Imaging of the cardiovascular ECM is essential in order to understand pathogenesis of diseases, such as atherosclerosis. Recently, extensive work has been performed on Raman microscopy imaging of vascular tissues, including aorta and heart tissues (Manoharan et al., 1992). Using Raman and fluorescence spectral microscopy, one study examined the composition of ceroid *in situ* in aorta and coronary artery plaques (Haka et al., 2011). The combination of fluorescence and Raman spectroscopy allowed for identification of ceroid via its fluorescence signature and elucidation of its chemical composition using Raman spectroscopy. Reference chemicals, including collagen, used to extract quantitative information about the tissue. In another study, Votteler et al. (2012) employed NIR Raman spectroscopy on the ECM of heart valves to detect degrees of collagen degradation. In a Raman imaging study, You et al. (2017) used 785 nm confocal Raman microscopy imaging and VCA to elucidate the differences in medial aortic calcification and atherosclerosis. They identified the major ECM macromolecules of the aorta (i.e., elastin/collagen) and quantified the spatial distribution of minerals and lipids across the wall thickness (Figure 4). Further, this study found that whitlockite was highly associated with calcification of the aorta. This study highlighted that confocal Raman microscopy in combination with spectral unmixing is particularly advantageous for imaging the complex layers of vascular tissues.

### Extracellular Matrix Deposition in Tissue Engineering

In recent years, Raman spectroscopy has served as a growing tool in the field of tissue engineering (TE) (Ember et al., 2017; Querido et al., 2017). TE represents an exciting multidisciplinary field that aims to generate live, functional replacement tissues (e.g., cardiac, bone, connective tissues) to treat a range of pathological disorders. A central challenge of TE is the generation of tissues that can recapitulate native ECM organizations in order to optimize functionality and performance upon native implantation (O'Connell et al., 2012). In this context, Raman spectroscopy can serve as an important quality assessment tool. Raman microscopy imaging can measure ECM distributions in engineered tissue sections in order to assess and guide the development of TE protocols. Further, fiber-optic Raman spectroscopic probes can serve as a clinical analytical tool, allowing for non-destructive, online monitoring of engineered tissue growth. Here, fiber-optic probes can be employed *in vivo* to monitor the development of engineered tissues after they have been implanted in the native environment. Further, probes can be employed *in vitro* to monitor the development of tissues during initial culture phases, allowing clinicians to select high-quality engineered tissue for implantation.

**Figure 5:**
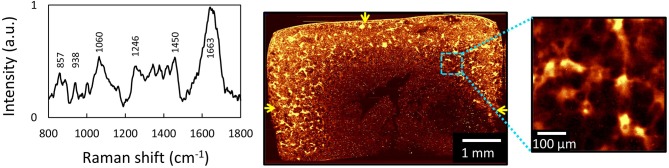
[Reprinted and modified with permission from Albro et al. (2018)] **(A)** Raw spectra of engineered cartilage tissue from chondrocyte-seeded agarose hydrogel scaffolds. Raman spectroscopic image (univariate analysis) of ECM heterogeneities in 56-day cultured large engineered cartilage tissue construct (Ã 6 × 3 mm) for **(B)** the full cross-section (10 μm spatial resolution) and **(C)** a localized peripheral region (1 μm resolution). Arrows represent media-exposed surfaces.

In a study measuring heterogeneities of ECM in engineered cartilage, we used 532 nm confocal Raman microscopy to image the distribution of newly deposited ECM in chondrocyte-seeded agarose-based cartilage tissue constructs (Figure 5). Here, we demonstrated that Raman imaging can achieve highly accurate, semi-quantitative measurements of the spatial distribution of GAG and collagen throughout engineered cartilage tissues, as evidenced by excellent agreement with profiles independently acquired via direct biochemical assaying of discrete spatial tissue sections. This validation serves as a foundational support for the exceptional quantitative nature of Raman image acquisitions. Further, in this study, Raman images were used to quantify the microstructural heterogeneity of ECM in engineered cartilage, allowing for the generation of quantitative comparisons of the quality of local ECM distributions to that of native articular cartilage.

For investigations into Raman spectroscopy-based online monitoring of engineered tissue development, Kunstar et al. (2013) showed that ECM deposition in polymeric scaffolds could be assessed using Raman microscopy. The authors identified specific markers corresponding to collagen and sulfated GAGs. Collagen synthesis was found to differ between chondrocyte-seeded scaffolds generated from isolated cells and cellular microaggregates. Normalized band-area ratios for collagen content of isolated cell-seeded samples gradually decreased during a 21-day culture period, whereas collagen content of the microaggregate-seeded samples increased significantly. Further, Kunstar et al. (2013) also implemented a fiber-optic Raman probe configuration to collect Raman spectra from multiple pores inside scaffolds. This highlights the exciting avenue of fiber-optic Raman spectral measurements for achieving highly valuable, non-destructive assessments of growing engineered tissues.

**Figure 6:**
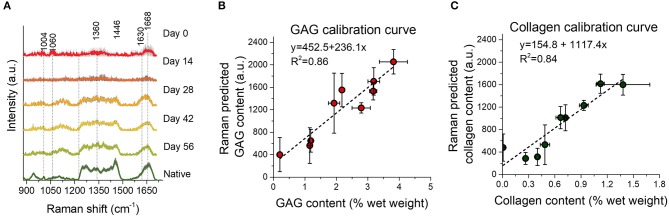
Reprinted and modified with permission from Bergholt et al. (2017). Biochemically measured GAG and collagen contents of TE constructs after 0, 14, 28, 42, and 56 days of culture. **(A)** Mean Raman spectra ± 1 standard deviation (SD) of native cartilage and TE constructs after varying culture durations. **(B,C)** Correlations between the Raman prediction (in arbitrary units) and biochemically measured GAG and collagen, respectively, with linear fits to the data. Raman spectroscopy showed an excellent correlation with biochemical assays for measurement of collagen (R^2^ = 0.84) and GAG (R^2^ = 0.86).

In a subsequent study, we demonstrated the ability of an NIR excitation fiber-optic Raman probe to perform online measurements of the deposition of collagen and GAG during the *in vitro* culture phase of engineered cartilage (Figure 6). Here, we measured diffuse NIR fiber-optic Raman spectra from living chondrocyte-seeded agarose tissue constructs in culture over a 56-day period. MCR modeling enabled quantitative biochemical analysis of the TE constructs. Raman spectroscopy showed an exceptional correlation with direct biochemical assays for measurements of collagen (R^2^ = 0.84) and GAG (R^2^ = 0.86). Further, Raman measured ECM contents exhibited a strong correlation with construct mechanical properties, suggesting that these non-destructive assessments can be clinically implemented to predict tissue functionality. Encouragingly, this study further confirmed that exposure of live tissue constructs to high intensity 785 nm excitation light induced no loss of cell viability or depression of ECM biosynthesis rates, serving as critical support for the applicability of NIR Raman spectroscopy in live-tissue quality control assessments.

In another recent study, Marzi et al. (2019) used NIR Raman spectroscopy to image human smooth muscle cells in cardiovascular tissue engineering. They monitored ECM remodeling in tissue-engineered rings comprised of human smooth muscle cells (SMC). Further, they measured the phenotypic switching in cultured SMCs and identified the impact of different culture conditions on ECM remodeling in the tissue-engineered ring constructs. Altogether, there is broad and growing utility of Raman spectroscopy in the field of TE.

### Collagen Structural Assessments

A major untapped potential of Raman spectroscopy is the development of techniques to measure subtle changes to the molecular structure of ECM constituents (e.g., denaturing, crosslinking) that are not readily discernible with immunological techniques. Perhaps the collagen matrix is the most intriguing ECM component from this perspective due to its presence in a complex arrangement of varying subtypes, alignment, secondary structure, and cross-linking states (Shoulders and Raines, 2009). These arrangements give rise to unique ECM properties that strongly modulate tissue functionality. As collagen organizational remodeling is a hallmark feature of many pathologies, the ability to assess these organizational differences is critical for Raman microscopy and *in vivo* diagnostic applications. Raman spectral techniques for discerning collagen organization are of growing interest, particularly due to the lack of robust biochemical analytical techniques that directly measure these organizational arrangements. In TE applications, Raman imaging of collagen can be used to identify the collagen subtypes being deposited in the ECM and, as such, serve to assess engineered tissue quality. For example, in cartilage TE, one would be able to measure the ratio of type-II to type-I collagen as a means of assessing the formation of hyaline vs. fibrous cartilage.

The collagen protein itself has a unique Raman spectrum that can easily be discriminated from most other proteins (You et al., 2017; Martinez et al., 2019) due to its fibrillar nature, as well as the abundance of collagen-specific amino acids, such as hydroxyproline. However, discrimination between collagens of varying subtypes, secondary structure, cross-linking states, and alignment represents a considerable challenge in Raman assessments due to the subtle influence of these factors on the collagen spectral signature. Several studies have begun to explore this analytical capacity. For one, it has been shown that there are significant differences in the amide band between Type I and Type IV collagen (Nguyen et al., 2012). Dehring et al. (2006) demonstrated that changes in the alpha-helical stability of type-I collagen from aging can be assessed by the area ratio of the amide III bands in the 1,235 cm^−1^ position. Gamsjaeger et al. (2017) demonstrated that analysis of the Raman band 1,660 cm^−1^ wavenumbers can be used to assess pyridinoline crosslinks in animal tissue models. Fields et al. (2017) implemented a technique of high temperature Raman spectroscopy to assess the primary polypeptide structure of different collagen subtypes. Further, recent work has demonstrated that the utilization of Raman spectral measurements with polarized lasers can be used to measure the critical parameter of collagen alignment or anisotropy (Lim et al., 2011; Bergholt et al., 2016a). Overall, these recent characterizations have introduced the important potential for Raman spectroscopy to serve as a robust and comprehensive tool for providing collagen structural analysis in biological ECMs. However, the widespread adoption of Raman spectroscopy for collagen structural analysis will require expanded efforts to develop robust multivariate analytical models, and identify appropriate experimental model systems to validate the molecular specificity of Raman measurements.

## Advanced Raman Methods

### Non-linear Raman Imaging

Several advanced Raman imaging approaches have emerged, such as coherent anti stokes Raman spectroscopy (CARS) and stimulated Raman scattering (SRS) microscopy (Camp et al., 2014; Schie et al., 2015; Lu et al., 2016). These techniques are able to speed up image acquisition times relative to conventional Raman spectroscopy, but require the implementation of complex instrumentation, including multiphoton lasers. Examples of application to ECM imaging are numerous. For instance, Mansfield et al. (2013) applied stimulated Raman scattering (SRS) in order to image the interface of cartilage and bone with high molecular contrast. SRS represents an advanced Raman technique that offers high speed imaging and reveals high quality spectroscopic images of the peri- and extracellular matrix. Since Raman is highly sensitive to lipids, the SRS approach revealed highly localized lipids in the pericellular matrix of chondrocytes. Recent exciting implementations of CARS and SRS enable the full range of Raman spectra to be measured, opening the opportunities for rapid hyperspectral Raman imaging (Parekh et al., 2010). Very recently, more exotic non-linear Raman techniques have emerged aiming toward label-free super-resolution imaging (Silva et al., 2016; Gong et al., 2019).

### Correlative Raman Spectroscopy and Imaging

To complement and potentially advance Raman spectroscopic imaging beyond conventional analysis, correlative imaging is continually advancing. Raman imaging has been combined with a plurality of other techniques, such as fluorescence (Bennet et al., 2014), atomic force microscopy (Brauchle et al., 2018), nanoindentation (Bergholt et al., 2016a), mass spectrometry (Bergholt et al., 2018), optic coherence tomography (OCT) (Patil et al., 2011), and FT-IR imaging (Lasch and Noda, 2017). Raman spectroscopy has also been correlated with mechanical measurement techniques (Unal and Akkus, 2015). Among the advanced techniques, a vibrant and promising field is the implementation of heterospectral imaging approaches, which directly interrelate the hyperspectral data of several modalities at the pixel level for a more comprehensive biomolecular analysis (Lasch and Noda, 2017, 2019; Bergholt et al., 2018; Ryabchykov et al., 2018). For instance, Lasch and Noda (2017) and Lasch and Noda (2019) introduced multimodal FT-IR, Raman, and MALDI-TOF imaging for tissue characterization. An important part of correlative imaging is the lateral resolution matching between diffraction limited Raman imaging and the correlative modality. This resolution matching can efficiently be achieved through binning or interpolation (Lasch and Noda, 2017; Bergholt et al., 2018).

### Fiber-Optic Raman Spectroscopy

The capability of Raman spectroscopy to offer label-free tissue characterizations has fuelled the field of fiber-optic Raman spectroscopy for clinical cancer detection and diagnostics (Shim et al., 2000; Utzinger and Richards-Kortum, 2003; Bergholt et al., 2014; Baker et al., 2018; McGregor et al., 2018). There have been extensive efforts in the development of miniaturized fiber-optic probes compatible with medical endoscopes in order to measure the biomolecular composition of tissues *in vivo* (Shim et al., 2000; Komachi et al., 2006). Since the clinical regulation pathway for label-free technologies is short relative to studies using exogenous contrast agents, there have been several large-scale human trials for *in vivo* tissue characterization and diagnostics. Key biomedical applications range from the *in vivo* diagnosis of diseases (Chau et al., 2008; Kendall et al., 2009; Pence et al., 2017) to the monitoring of the growth of engineered tissues (Bergholt et al., 2017). These techniques, in general, probe both the cells and ECM, and offer a compound Raman spectrum that carries information about the overall tissue composition. As an example of targeted *in vivo* analysis, the Mahadevan group recently performed an *in vivo* study of impaired cervical remodeling in a mouse model of delayed parturition (O'Brien et al., 2017). Deletion of cyclooxygenase-1 (Cox-1) results in delayed parturition in mice. They used Raman spectroscopy to detect spectral changes related to extracellular matrix proteins, lipids, and nucleic acids over pregnancy. They concluded that *in vivo* Raman spectroscopy non-invasively detected abnormal remodeling in the Cox-1 null mouse, and clearly demonstrated that the cervix plays a key role in their delayed parturition.

### Prospective and Conclusion

Histological imaging has long served as the gold standard for visualizing specific molecular ECM constituents. Raman spectroscopy represents a label-free tool that offers an additional level of molecular information, with applicability for both microscopy-based characterizations and minimally invasive diagnostics of the ECM structure and composition.

There are still many challenges in the widespread adoption of Raman spectroscopy imaging in biomedicine, predominantly due to the technical difficulties in data interpretation and analysis. Mounting efforts must, therefore, be placed into streamlining Raman analysis through the development of protocols for correlative Raman spectroscopy and imaging. Further, more standardized preprocessing, data analysis techniques, and calibration transfer are needed in order to improve the reproducibility of results across laboratories. Furthermore, for Raman spectroscopic imaging to gain wider acceptance in biomedicine, Raman imaging of tissues should be correlated with appropriate reference methods, such as immunofluorescence or direct biochemical assay measurements. Future developments could include correlation of hyperspectral Raman images of the ECM with sophisticated molecular-based characterization approaches, such as proteomics.

In terms of analysis, besides conventional multivariate algorithms, we anticipate that Raman spectroscopy will begin to rely heavily on deep learning approaches that still remain in their early stages for Raman spectral analysis. Since deep learning relies on neural networks, the large datasets that Raman imaging provides could serve as an excellent data source. The use of deep learning can potentially be used for regression and classification, but also for Raman image resolution enhancement and correlative imaging.

Overall, Raman spectroscopy represents a powerful tool that provides a wealth of quantitative information about the molecular structure of a tissue's ECM. The potential application avenues for Raman spectroscopy in biomedicine are vast. In many respects, the field is still in its infancy and will continue to grow with the expansion of equipment technologies and analytical algorithms. We anticipate that in the near future, Raman spectroscopy can develop into one of the premiere technologies for tissue diagnostics and analytics.

## Author Contributions

All authors listed have made a substantial, direct and intellectual contribution to the work, and approved it for publication.

### Conflict of Interest

The authors declare that the research was conducted in the absence of any commercial or financial relationships that could be construed as a potential conflict of interest.
